# *ERBB2* exon 20 insertions are rare in Brazilian non‐small cell lung cancer

**DOI:** 10.1111/1759-7714.14605

**Published:** 2022-10-17

**Authors:** Rodrigo de Oliveira Cavagna, Beatriz Garbe Zaniolo, Flávia Escremim de Paula, Gustavo Noriz Berardinelli, Iara Santana, Eduardo Caetano Albino da Silva, Josiane Mourão Dias, Alexandre Arthur Jacinto, Rachid Eduardo Noleto da Nóbrega Oliveira, Pedro de Marchi, Letícia Ferro Leal, Rui Manuel Reis

**Affiliations:** ^1^ Molecular Oncology Research Center Barretos Cancer Hospital Barretos Brazil; ^2^ Barretos School of Health Sciences Dr. Paulo Prata – FACISB Barretos Brazil; ^3^ Molecular Diagnostic Laboratory Barretos Cancer Hospital Barretos Brazil; ^4^ Department of Pathology Barretos Cancer Hospital Barretos Brazil; ^5^ Department of Medical Oncology Barretos Cancer Hospital Barretos Brazil; ^6^ Deparment of Radiology Barretos Cancer Hospital Barretos Brazil; ^7^ Deparment of Thoracic Surgery Barretos Cancer Hospital Barretos Brazil; ^8^ Oncoclinicas Rio de Janeiro Brazil; ^9^ Life and Health Sciences Research Institute (ICVS), School of Medicine, University of Minho Braga Portugal; ^10^ ICVS/3B's – PT Government Associate Laboratory Braga/Guimarães Portugal

**Keywords:** Brazil, *ERBB2*, exon 20 insertions, non‐small cell lung cancer

## Abstract

*ERBB2* exon 20 insertions may impact the clinical management of lung cancer patients. However, the frequency of *ERBB2* exon 20 insertions in lung cancer patients in Brazil is scarce. Here, we analyzed 722 Brazilian non‐small cell lung cancer (NSCLC) patients from Barretos Cancer Hospital that were indicated to require routine lung cancer molecular testing. *ERBB2* exon 20 insertions were evaluated by a targeted panel using next‐generation sequencing (NGS). Clinicopathological and molecular data were collected from patient medical records. Among the 722 NSCLC patients, 85.2% had lung adenocarcinomas, 53.9% were male, 66.8% were quitter or current smokers, and 63.2% were diagnosed at an advanced stage of the disease. We identified 0.8% (6/722) of patients who harbored the insertion p.(Tyr772_Ala775dup) at exon 20 of the *ERBB2* gene. All *ERBB2* mutated patients were diagnosed with lung adenocarcinoma, were never smokers, and wild‐type for *EGFR, KRAS*, and *ALK* hotspot alterations. Less than 1% of Brazilian NSCLC patients harbor *ERBB2* exon 20 insertions, yet they could benefit in future from the new drugs in development.

## Graphical abstract

ERBB2 exon 20 insertions are rare in Brazilian patients and mutually exclusive with EGFR, KRAS, and ALK alterations.

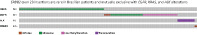

## INTRODUCTION

Lung cancer remains the deadliest cancer worldwide.^1^ The International Agency for Research on Cancer estimated about 2.2 million new cases and 1.8 million deaths to the year 2020 around the world.^1^, ^2^ In Brazil, lung cancer is one of the most frequent diagnosed cancers, and the leading cause of cancer‐related deaths.^1^, ^2^

Targeted therapies towards specific molecular alterations in non‐small cell lung cancer (NSCLC) tumors, such as *EGFR* mutations and *ALK* translocations, have improved the clinical management and prognosis for patients.^3^ The human epidermal growth factor receptor 2 gene (*ERBB2*) is a proto‐oncogene that has emerged as a candidate for targeted therapies in lung adenocarcinoma patients.^4^ *ERBB2* gene is amplified in about 2% of NSCLC tumors, and *ERBB2* mutations have been reported in 2%–4% of NSCLC tumors.^4^ Most *ERBB2* mutations are duplications or insertions of 12 nucleotides at the exon 20.^4^ This insertion adds four aminoacids (TyrValMetAla) in the kinase domain at codon 775, leading putatively to increased gene activation.^4^, ^5^, ^6^ Additionally, *ERBB2* exon 20 insertions are mutually exclusive with *EGFR* and *KRAS* mutations as either *ALK* translocations.^4^, ^5^, ^6^ A recent comprehensive review highlighted the importance of *ERBB2* mutations in the clinical management of NSCLC patients, and several drugs targeting *ERBB2* insertion in exon 20, such as trastuzumab deruxtecan, poziotinib and pyrotinib, have been under clinical trials.^6^

The frequency of *ERBB2* exon 20 insertions in lung cancer patients in Brazil is scarce. Therefore, we aimed to evaluate the frequency of *ERBB2* exon 20 insertions and their clinicopathological and molecular features in a series of Brazilian NSCLC patients.

## METHODS

We evaluated a retrospective series of 722 Brazilian NSCLC patients, coming from almost all Brazilian regions (Supplementary Figure 1), diagnosed at Barretos Cancer Hospital (Barretos, São Paulo, Brazil). All patients were indicated to routine lung cancer molecular testing (*EGFR*, *KRAS*, and *ERBB2*) and *ALK* translocations at the Department of Molecular Diagnosis from the institution between the years 2018 and 2021. The clinicopathological and molecular data were collected from the medical records of patients. The present study was approved by the Barretos Cancer Hospital IRB (project no. 630/2012) and waived written informed consent due to the retrospective nature of the study. All procedures were performed following the Declaration of Helsinki.

DNA was isolated from formalin‐fixed paraffin‐embedded (FFPE) tumor using the commercial kit QIAamp DNA Micro Kit (Qiagen). DNA concentration and purity were evaluated by Nanodrop 2000 (Thermo Scientific) and by Qubit 2.0 Fluorometer (Thermo Fisher Scientific) with Qubit dsDNA HS assay kit (Thermo Fisher Scientific).

The mutational status of the exon 20 from *ERBB2* gene (NM_004448) was assessed by next‐generation sequencing using the targeted panel TruSight Tumor 15 (Illumina) on the MiSeq instrument, according to the manufacturer's instructions. For the read alignment and variant calling, we used the BaseSpace BWA Enrichment version 2.1 (Illumina) and the Sophia DDM software version 4.2 (Sophia Genetics SA). Only *ERBB2* insertions in exon 20 with depth higher than 500x and allele frequency higher than 5% were selected.

Detection of *ALK* rearrangements were routinely performed in FFPE sections using Ventana ALK (D5F3) CDx Assay (Roche) according to the manufacturer's instructions on automated equipment. Slides were evaluated by a specialist pathologist. *ALK* rearrangement was defined as positive by the presence of strong granular cytoplasmic staining in tumor cells (any percentage).

For statistical analysis, we used the frequency and percentage to describe categorical variables and median to describe continuous variables, using the software IBM SPSS Statistics Version 22 (IBM Corp).

## RESULTS

The clinicopathological and molecular features of the 722 Brazilian NSCLC patients is summarized in Table 1. Among NSCLC patients, 85.2% (*n* = 615/722) had lung adenocarcinomas, 1.9% (*n* = 14/722) had squamous cell carcinoma, and 12.9% (*n* = 93/722) were from other NSCLC histology. The median age was 64.0 years old, and 53.9% were male (*n* = 389/722). Concerning tobacco use, 66.8% (*n* = 482/722) were quitters or current smokers, 53.6% (*n* = 387/722) were diagnosed with a performance status of 0/1, 46.6% (*n* = 336/722) presented with loss of weight 6 months prior the diagnosis, and 63.2% (*n* = 456/722) were diagnosed with advanced stage of the disease.

**TABLE 1:** Clinicopathological and molecular features of NSCLC patients

Variable	Category	NSCLC patients (*n* = 722)	
		N	(%)
**Age (years)** ^**a**^	Median (range)	64.0 (26–94)	
	≤64	373	51.7%
	>64	349	48.3%
**Sex**	Female	333	46.1%
	Male	389	53.9%
**Tobacco use**	Never smoker	177	24.5%
	Quitter smoker	249	34.5%
	Current smoker	233	32.3%
	Missing	63	8.7%
**Loss of weight** ^**b**^	No	216	29.9%
	Yes ≤10% of weight	194	26.9%
	Yes >10% of weight	142	19.7%
	Missing	170	23.5%
**ECOG PS**	0	111	15.4%
	1	276	38.2%
	2	140	19.4%
	3/4	74	10.2%
	Missing	121	16.8%
**Histology**	Adenocarcinoma	615	85.2%
	Squamous cell carcinoma	14	1.9%
	Others^c^	93	12.9%
**Stage at diagnosis** ^**d**^	I/II	28	3.9%
	III	154	21.3%
	IV	456	63.2%
	Missing	84	11.6%
**Metastasis at diagnosis**	No	182	25.2%
	Yes, central nervous system	146	20.2%
	Yes, others	273	37.8%
	Missing	121	16.8%
***EGFR* status**	Wild‐type	559	77.4%
	Mutant	163	22.6%
***KRAS* status**	Wild‐type	533	73.8%
	Mutant	189	26.2%
***ERBB2* exon 20 insertions**	Wild‐type	716	99.2%
	Mutated	6	0.8%
***ALK* status**	Wild‐type	621	86.0%
	Mutant	38	5.3%
	Missing/inconclusive	63	8.7%
**Vital status**	Alive	336	46.5%
	Death	352	48.8%
	Missing	34	4.7%

n, number of patients.

^a^ Age was categorized into two groups considering the median of the entire series as the cutoff.

^b^ Last 6 months before the diagnosis.

^c^ Adenosquamous, NOS (not otherwise specified), large cell, sarcomatoid carcinoma, and neuroendocrine carcinoma.

^d^ According to AJCC seventh edition.

Molecularly, 26.2% (*n* = 189/722) were *KRAS*‐mutated, 22.6% (*n* = 163/722) were *EGFR*‐mutated, and 5.3% (*n* = 38/722) were *ALK*‐translocated (Table 1). *ERBB2* exon 20 insertions were identified in 0.8% (*n* = 6/722) of all patients.

**FIGURE 1:**
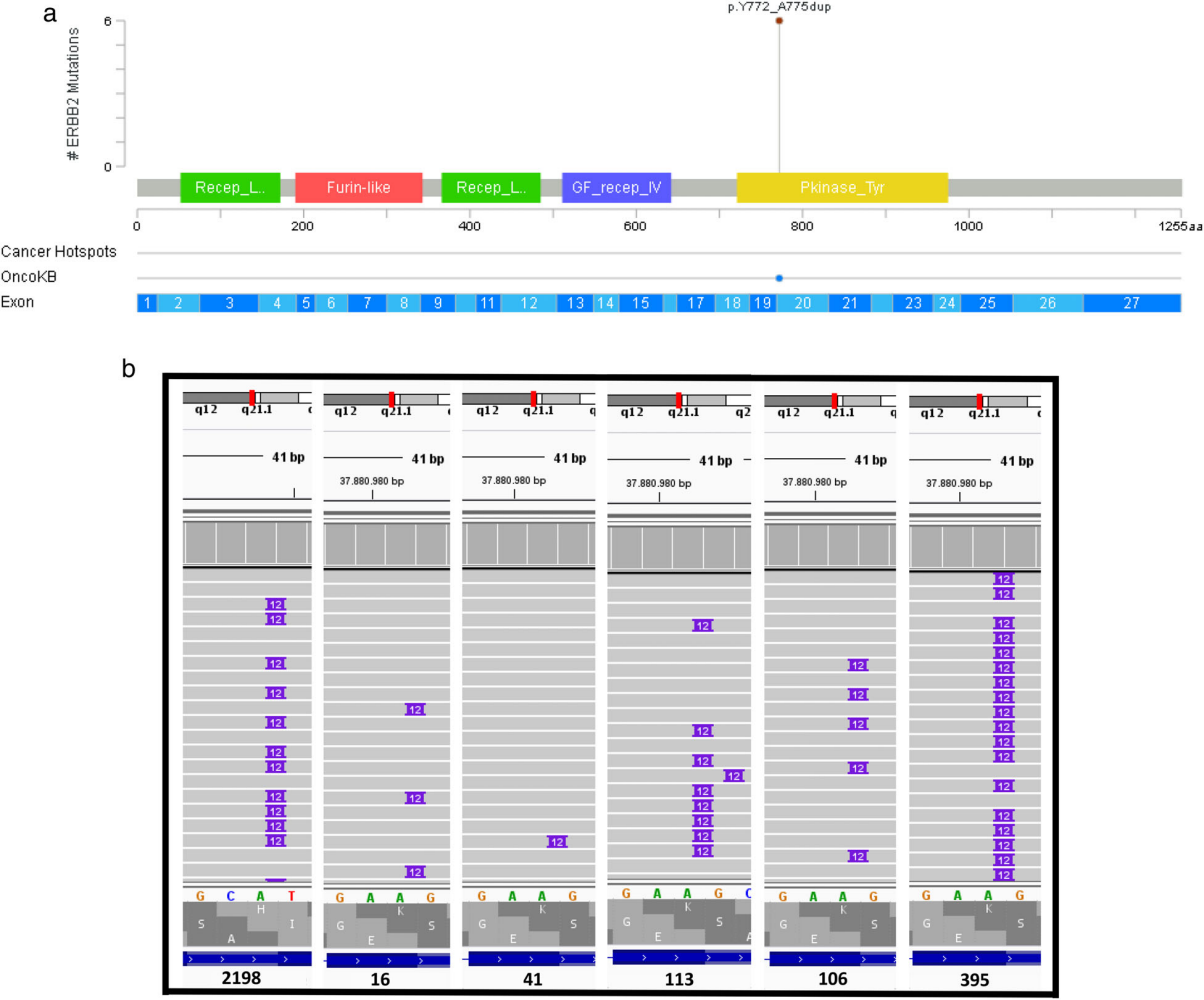
(a**)** *ERBB2* exon 20 insertions indentified in non‐small cell lung cancer (NSCLC) patients (*n* = 6); Created in cbioportal.gov. (b) *ERBB2* exon 20 insertions identified observed in IGV software

**FIGURE 2:**
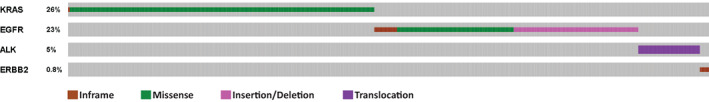
Representative molecular alterations in non‐small cell lung cancer (NSCLC) patients (*n* = 722). Only mutant patients are shown (*n* = 396)

We identified six lung adenocarcinoma patients harboring the *ERBB2* inframe insertion p.(Tyr772_Ala775dup) in exon 20 (Figure 1a/b and Table A.1). The features of mutant *ERBB2* patients are described in Table 2. Most patients were female, never smokers, and diagnosed at an advanced stage of disease with metastasis of lung/pleura, bone, and lymph node. All six patients were diagnosed with lung adenocarcinoma and were wild‐type for *EGFR, KRAS* and *ALK‐*translocations (Table 2; Figure 2).

**TABLE 2:** Clinicopathological and molecular features from *ERBB2*‐mutant patients (*n* = 6)

Variable	*ERBB2*‐mutant patients p.(Tyr772_Ala775dup)					
	ID‐113	ID‐16	ID‐395	ID‐41	ID‐2198	ID‐106
**Age (year)**	75.0	75.0	66.0	52.0	61.0	58.0
**Sex**	Female	Male	Female	Female	Male	Female
**Tobacco Use**	Never smoker	Never smoker	Never smoker	Never smoker	Missing	Never smoker
**Loss of weight** ^**a**^	Yes, <= 10%	No	Missing	Yes, <= 10%	No	No
**ECOG PS**	2	Missing	3	1	Missing	1
**Histology**	Adenocarcinoma	Adenocarcinoma	Adenocarcinoma	Adenocarcinoma	Adenocarcinoma	Adenocarcinoma
**stage at diagnosis** ^**b**^	IV	IV	IV	IV	II	IV
**Metastasis at diagnosis**	Lung / pleura	Lung / pleura, Bone, lymph node	Bone	Lung / pleura	No	Liver, lymph node
***EGFR* status**	Wild‐type	Wild‐type	Wild‐type	Wild‐type	Wild‐type	Wild‐type
***KRAS* status**	Wild‐type	Wild‐type	Wild‐type	Wild‐type	Wild‐type	Wild‐type
***ALK* status**	Wild‐type	Wild‐type	Wild‐type	Wild‐type	Missing	Wild‐type
**Vital status**	Alive‐active disease	Alive‐active disease	Death‐cancer	Death‐cancer	Alive‐active disease	Death‐cancer
**Overall survival (months)**	27.8	27.2	3.5	28.6	2.8	2.0

^a^ Last 6 months before the diagnosis.

^b^ According to AJCC seventh edition.

## DISCUSSION

Molecular *ERBB2* therapies, namely targeting exon 20 insertion, are being explored in NSCLC patients.^4^ Herein, we report the frequency of *ERBB2* exon 20 insertions in a series of 722 Brazilian NSCLC patients.

The frequency of NSCLC patients harboring *ERBB2* mutations ranges from 2%–5% in the literature.^6^, ^7^, ^8^ The Cancer Genome Atlas (TCGA) reported a frequency of 2.6% (*n* = 6/230) for *ERBB2*‐mutated patients in lung adenocarcinomas.^7^ Concerning only *ERBB2* exon 20 insertions, TCGA reported a frequency of 1.3% (*n* = 3/230).^7^ A recent comprehensive review on the topic reported that *ERBB2* exon 20 insertions are present in about 1.5% of NSCLC patients and account for 90% of *EBBB2* mutations.^6^ The study by Li et al. reported an *ERBB2* mutational frequency of 3% (*n* = 4/148) in lung adenocarcinomas patients – all identified mutations were insertions at exon 20.^8^ Recently, Carrot‐Zhang et al. analyzed *ERBB2* in Latin Americans (Mexico and Colombia patients), but no *ERBB2* exon 20 insertion was reported.^9^ Moreover, a Brazilian study using the Foundation One or Foundation ACT, described 5% of NSCLC patients harboring *ERBB2* mutations (*n* = 26/513), with 1.4% (7/513) exhibiting the exon 20 insertions (p. A775_G776INSYVMA), but no additional information was reported.^10^

Our study constituted a larger assessment of *ERBB2* exon 20 insertions in Brazilian patients. Only six out of 722 (0.8%) were mutated, being mainly female and never smokers, in agreement with studies from other geographic regions.^4^, ^6^, ^8^ Considering the frequency reported in our study and by Mascarenhas et al.,^10^ we may infer that about 1% of NSCLC patients harbor *ERBB2* exon 20 insertions in Brazil. As expected, *ERBB2* mutations were mutually exclusive with *EGFR, KRAS* and *ALK* alterations. In our study, all *ERBB2* exon 20 insertions were the p.(Tyr772_Ala775dup) inframe insertion. According to the review by Friedlaender et al., the most common *ERBB2* exon 20 insertions in NSCLC patients are p.(Tyr772dupTyrValMetAla) and the p.(Ala775_Gly776insTyrValMetAla).^6^ Due to the low number of *ERBB2* exon 20 insertion mutated patients in our study, no statistically significant association was performed.

Notably, we found six patients in our series harboring *ERBB2* exon 20 insertion mutations that could benefit from treatment with tyrosine kinase inhibitors (TKIs), such as poziotinib, pyrotinib, and trastuzumab deruxtecan. A phase II basket trial (ZENITH20) evaluated 90 patients harboring *ERBB2* exon 20 insertion mutations treated with poziotinib which showed an objective response rate (ORR) of 27.8% (95% CI: 18.9–38.2), with 25 of 90 patients achieving partial response, a disease control rate of 70% (95% CI: 59.4–79.2), and a median progression‐free survival (PFS) of 5.5 months (95% CI: 3.9–5.8). Patients pretreated with three or more prior treatment lines had greater responses (ORR, 37.1%).^11^ Another phase II trial study evaluated 60 advanced (IIIB–IV) lung adenocarcinoma patients harboring *ERBB2* mutations and previously treated with platinum‐based chemotherapy.^12^ In this study, 49 patients were identified with *ERBB2* exon 20 insertions (12‐bp insertion, *n* = 44; 9‐bp insertion, *n* = 5) and there were 11 patients with missense mutations.^12^ From the 60 patients, 18 showed an ORR (30%, 95% CI: 18.8%–43.2%), all with partial response, and a median PFS and overall survival of 6.9 months (95% CI: 5.5–8.3). In patients with different mutation types, the ORR was higher in 44 patients harboring 12‐ and 9‐bp exon 20 insertions (27.3% and 60.0%, respectively).^12^ Finally, the open‐label phase 2 DESTINY‐Lung01 study evaluated trastuzumab deruxtecan in 91 nonsquamous metastatic NSCLC patients harboring *ERBB2* mutations that relapsed during standard treatment or had refractory to standard treatment (platinum‐based chemotherapy, anti‐PD‐1 or anti‐PD‐L1 treatment), and reported an ORR in 55% of the patients.^4^

Importantly, *ERBB2* can be deregulated by other mutations, namely missense in other regions, and harbor gene amplification mechanisms,^6^, ^8^ which were not explored in the present study, and could lead to a higher percentage of *ERRB2* genomic alterations in Brazilian lung cancer patients.

In conclusion, we report that less than 1% of Brazilian NSCLC patients harbor the *ERBB2* exon 20 insertions. Nevertheless, they could putatively benefit from ERBB2 targeted therapies.

## CONFLICT OF INTEREST

The authors have nothing to disclose and confirm they have not received any grants or support related to this study.

## Supporting information


**Supplementary Figure 1** **–** Distribution of patients from Barretos Cancer Hospital (n = 722). Created using Microsoft Office Excel 2019.

**Table A.1** *ERBB2* exon 20 insertions molecular features.
