# Supplementary material for: ERBB2 exon 20 insertions are rare in Brazilian non‐small cell lung cancer
Source: Thorac Cancer. 2022 Oct 17;13(23):3402–7. doi: 10.1111/1759-7714.14605 (PMC9715798; doi:10.1111/1759-7714.14605)

Supplementary Figure 1 – Distribution of patients from Barretos Cancer Hospital (n=722). Created using Microsoft Office Excel 2019

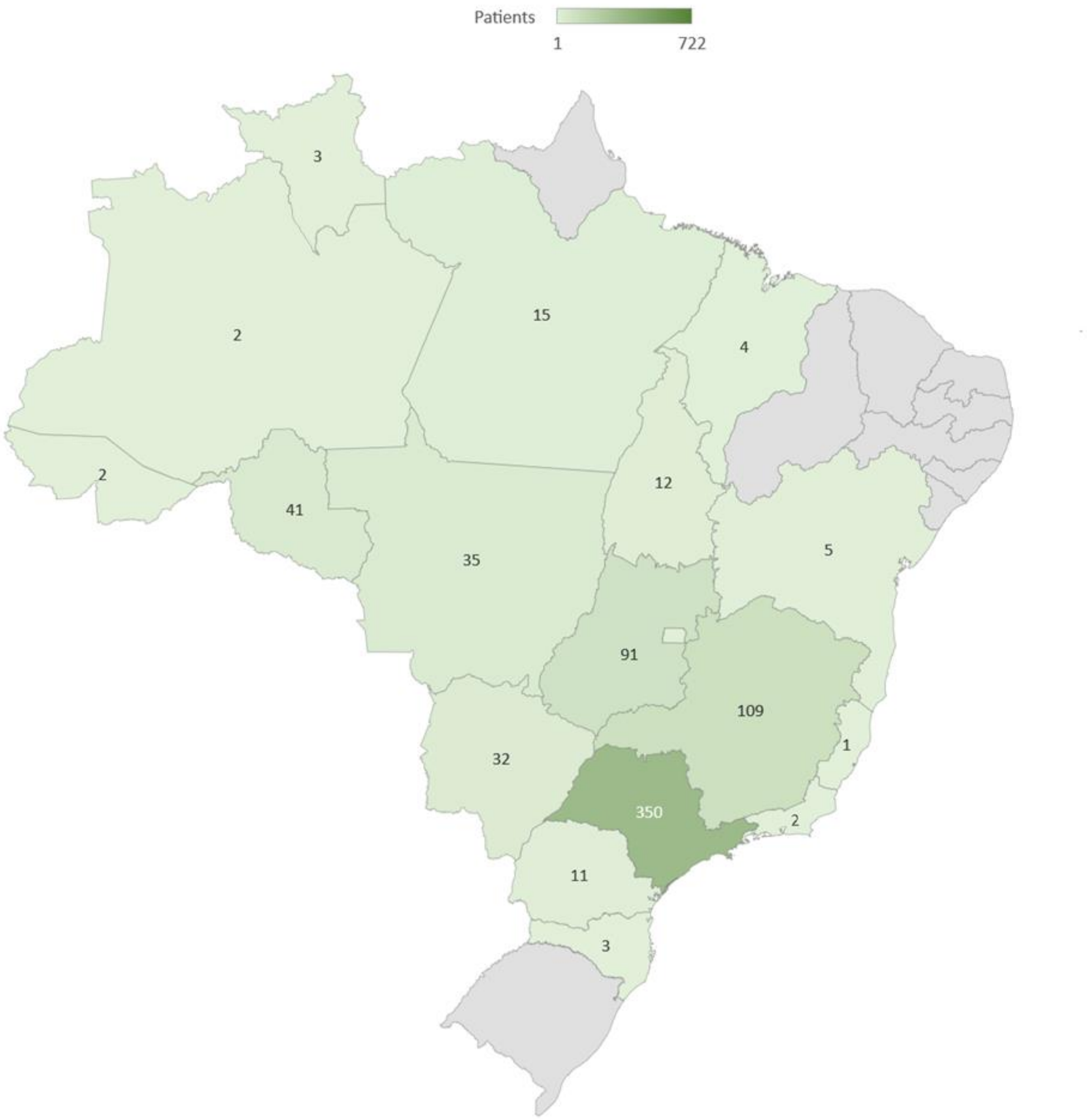

Supplement: Supplementary file 1 — Supplementary Figure 1 – Distribution of patients from Barretos Cancer Hospital (n = 722). Created using Microsoft Office Excel 2019. [file TCA-13-3402-s002.pdf]
