# Supplementary material for: ERBB2 exon 20 insertions are rare in Brazilian non‐small cell lung cancer
Source: Thorac Cancer. 2022 Oct 17;13(23):3402–7. doi: 10.1111/1759-7714.14605 (PMC9715798; doi:10.1111/1759-7714.14605)
Supplement: Supplementary file 2 — Table A.1 ERBB2 exon 20 insertions molecular features. [file TCA-13-3402-s001.docx]

**Table A.1 –***ERBB2* exon 20 insertions molecular features.

| **ID** | **c.** | **p.** | **VAF (%)** | **Depth** |
| --- | --- | --- | --- | --- |
| 16 | c.2313_2324dup | p.(Tyr772_Ala775dup) | 23.9 | 2506 |
| 41 | c.2313_2324dup | p.(Tyr772_Ala775dup) | 19.69 | 60787 |
| 106 | c.2313_2324dup | p.(Tyr772_Ala775dup) | 26.26 | 3058 |
| 113 | c.2313_2324dup | p.(Tyr772_Ala775dup) | 43.03 | 17767 |
| 395 | c.2313_2324dup | p.(Tyr772_Ala775dup) | 61.89 | 21439 |
| 2198 | c.2318_2319insAATGGCTTACGT | p.(Tyr772_Ala775dup) | 62.65 | 15583 |
